# Gender differences in clinical and biochemical parameters among patients hospitalized for schizophrenia: towards precision medicine

**DOI:** 10.1007/s00406-023-01644-4

**Published:** 2023-07-12

**Authors:** Cecilia Maria Esposito, Francesca De Cagna, Alice Caldiroli, Enrico Capuzzi, Alessandro Ceresa, Martina Di Paolo, Anna Maria Auxilia, Martina Capellazzi, Ilaria Tagliabue, Luisa Cirella, Massimo Clerici, Natascia Brondino, Jennifer L. Barkin, Pierluigi Politi, Massimiliano Buoli

**Affiliations:** 1 Department of Brain and Behavioral Sciences, University of Pavia, Pavia, Italy; 2 Department of Neurosciences and Mental Health, Fondazione IRCCS Ca’ Granda Ospedale Maggiore Policlinico, Via F. Sforza 35, 20122 Milan, Italy; 3 Department of Mental Health and Addictions, ASST Lariana, Como, Italy; 4 Psychiatric Department, Azienda Socio-Sanitaria Territoriale Monza, Monza, Italy; 5 Department of Medicine and Surgery, University of Milano Bicocca, Monza, Italy; 6 Healthcare Professionals Department, Foundation IRCCS Ca Granda Ospedale Maggiore Policlinico, Milan, Italy; 7 ASST Pavia, Pavia, Italy; 8 Mercer University School of Medicine, Macon, GA USA; 9 Department of Pathophysiology and Transplantation, University of Milan, Milan, Italy

**Keywords:** Schizophrenia, Gender, Clinical features, Biochemical markers

## Abstract

**Background:**

The scientific literature shows some gender differences in the clinical course of schizophrenia. The aim of this study is to identify gender differences in clinical and biochemical parameters in subjects affected by schizophrenia. This would allow for the implementation of individualized treatment strategies.

**Methods:**

We examined a large set of clinical and biochemical parameters. Data were obtained from clinical charts and blood analyses from a sample of 555 schizophrenia patients consecutively admitted for exacerbation of symptoms to the inpatient clinic of Fondazione IRCCS Policlinico (Milan) or ASST Monza in Italy from 2008 to 2021. Univariate analyses, binary logistic regression, and a final logistic regression model were performed with gender as dependent variable.

**Results:**

The final logistic regression models showed that male patients (compared to females) were more prone to lifetime substance use disorders (p = 0.010). However, they also had higher GAF (global functioning) mean scores (p < 0.001) at the time of hospitalization. Univariate analyses showed that male patients (with respect to females) had an earlier age at onset (p < 0.001), a more frequent family history of multiple psychiatric disorders (p = 0.045), were more often smokers (p < 0.001), had a more frequent comorbidity with at least one psychiatric disorder (p = 0.001), and less often suffered from hypothyroidism (p = 0.011). In addition, men had higher levels of albumin (p < 0.001) and bilirubin (t = 2.139, p = 0.033), but lower levels of total cholesterol (t = 3.755, p < 0.001).

**Conclusions:**

Our analyses indicate a less severe clinical profile in female patients. This is evident especially in the early years of the disorder, as suggested by less comorbidity with psychiatric disorders or later age at onset; this is consistent with the related literature. In contrast, female patients seem to be more vulnerable to metabolic alterations as demonstrated by more frequent hypercholesterolemia and thyroid dysfunction. Further studies are needed to confirm these results in the framework of precision medicine.

## Introduction

Schizophrenia is a serious mental disorder characterized by the presence of pervasive psychotic symptoms and progressive functional decline [1]. Its lifetime prevalence is approximately 7.2 per 1.000 worldwide [2] and there is considerable associated disability and cost [3]. Notably, delayed treatment worsens the prognosis of schizophrenia [4] and early, effective intervention is recommended for affected individuals [5, 6].

Early age at onset [7, 8], duration of illness [9], prominent negative [10] and cognitive symptoms [11], psychiatric [12] and medical comorbidities [13], and male gender [14] were identified as factors associated with severity and poor response to antipsychotics in schizophrenia. Gender is particularly important in the management of different psychiatric disorders, either because some psychiatric conditions are more prevalent in one of the two genders [15] or because female/male gender is associated with a different severity of illness or clinical presentation (e.g. rapid cycling seems to be more prevalent in female bipolar patients) [16, 17]. Nevertheless, gender differences in mental health can be explained by several variables including the effects of sex hormones, a variable vulnerability to stress, gender-based violence, low self-esteem, belonging to a gender minority, and socioeconomic/family-related factors [18].

Even though schizophrenia does not present a different distribution between males and females [2], several clinical and biological differences were reported between female and male patients [19]. In general, women appear to have a later age at onset and higher premorbid functioning than men [19, 20]. Negative symptoms are predominant in male patients, while affective disturbances present more often in females [19]. Furthermore, men affected by schizophrenia are more prone to substance use disorders and suicidal behaviours, relative to women [21, 22]. Globally, women seem to have more favourable prognostic clinical factors at the onset of the disorder. However, this advantage seems to dissipate over time, perhaps due to the weakening of the protective role of oestrogens [14, 19]. This observation is also supported by the fact that women suffering from schizophrenia demonstrate greater vulnerability for development of physical problems (e.g. diabetes) which is, in turn, associated with increased mortality [23, 24].

Regarding biological factors, some data indicate that relatives of women affected by schizophrenia have a higher risk (with respect to men) to develop the disorder [25] and that specific genetic polymorphisms confer an increased risk of illness in only one gender [26]. As mentioned before, oestrogens seem to exert a protective role for mental health and women affected by schizophrenia were found to have subnormal oestrogen levels as well as hyperprolactinemia (suppressing the production of gonadal hormones) compared to healthy women [27]. In line with these findings, female schizophrenia patients seem to experience poorer sleep quality and more inflammation than males [28]. This may also explain their susceptibility to metabolic syndrome and other comorbidities [28]. In contrast, structural neuroimaging studies have shown enlarged ventricles and smaller frontal lobes in men (relative to women) with schizophrenia [29]. Consistently, male schizophrenia patients demonstrate different neurocognitive profiles than female patients. Specifically, female gender is associated with higher performance on tests of verbal learning and memory [30].

In the light of this evidence, the purpose of the present study is to identify gender differences in clinical variables and biochemical parameters in patients affected by schizophrenia. The results of the present study will support the development and implementation of targeted treatment strategies for schizophrenia patients.

## Materials and methods

A retrospective design was employed for this study. A total sample of 555 patients with a diagnosis of schizophrenia was enrolled. Patients that were hospitalized (consecutively) between 2008 and 2021 to the inpatient clinic of ASST Monza, Italy (N = 270) or Fondazione IRCCS Policlinico (N = 285) were enrolled. Concerning the patients who had multiple hospitalizations, we considered only the data regarding the last hospitalization. All patients were screened by an expert senior psychiatrist working in the inpatient clinic. The diagnosis of schizophrenia, as well as that of any psychiatric comorbidity, was performed according to DSM criteria via clinical interview [1]. If the patient met the criteria for more than one psychiatric diagnosis, schizophrenia was still the most clinically relevant diagnosis. Clinical and biological data were obtained from electronic medical records and intranet hospital applications. Routine blood analyses are generally conducted during the first day of hospitalization in the morning. After being discharged, patients were generally monitored in community mental health services, where they are monitored long-term.

The following inclusion criteria were used to define the sample: (1) age ≥ 18 years, (2) admission to inpatient services between 2008 and 2021, and (3) diagnosis of schizophrenia. Exclusion criteria were the following: (1) age ≤ 18 years, (2) pharmacological treatments that could favor the onset of psychotic symptoms (e.g. steroids and levetiracetam), (3) medical comorbidities that could significantly affect biochemical parameters (e.g. rheumatoid arthritis), and (4) peripartum (pregnancy to six months after delivery) as this period is characterized by biological changes that may precipitate an onset of psychotic symptom [31].

During admission to the inpatient clinic, we collected the following variables:

- age, gender, age at onset of schizophrenia, duration of untreated illness (measured in years) (defined as the time between the onset of the disorder and the prescription of first antipsychotic [4]), duration of illness (years), duration of the current hospitalization (days), number of lifetime hospitalizations, lifetime history of substance or alcohol misuse disorders, presence of lifetime history of poly-substance use disorders, family history for single or multiple psychiatric disorders, family history for substance use disorders, main antipsychotic treatment at the time of hospitalization, poly-antipsychotic treatment at the time of admission, comorbidity with at least one psychiatric diagnosis, multiple psychiatric comorbidity, comorbidity with personality disorders, presence of lifetime suicidal attempts (defined as self-harm combined with the intent to die [32]), number of lifetime suicidal attempts, smoking status, number of cigarettes/day, medical comorbidity, comorbidity with hypothyroidism, comorbidity with diabetes, comorbidity with obesity (defined as a body mass index (BMI) ≥ 30 [33]), comorbidity with hypercholesterolemia, multiple medical comorbidities, current treatment with statins, current treatment with thyroid hormone, lifetime psychotherapeutic treatment, scores of Global Assessment of Functioning (GAF: measure of patient’s global functioning, where scores range from 1 to 100 and higher scores indicate less impairment [34]) and the Positive and Negative Syndrome Scale score (PANSS: measure of the severity of positive and negative symptoms as well as of general psychopathology of schizophrenia [35], with a score ranging from 30 to 210 and a score ≥ 58, 75, 95 and 116 accounting respectively for mild, moderate, marked and severe presentation of illness).
- The biochemical parameters collected were number of red blood cells (RBC) (10^12/L), mean corpuscular volume (MCV) (fL), haemoglobin (HB) (g/dL), number of white blood cells (WBC) (10^9/L), number of lymphocytes (10^9/L), number of neutrophils (10^9/L), neutrophil/lymphocyte ratio (NLR), number of platelets (PLT) (10^9/L), mean platelet volume (MPV) (fL), pseudocholinesterase (PCHE) (U/L), total plasmatic proteins (g/dL), albumin (g/dL), bilirubin (mg/dL), uric acid (mg/dL), cholesterol (mg/dL), low-density lipoproteins (LDL) (mg/dL), high-density lipoproteins (HDL) (mg/dL), glycaemia (mg/dL), creatine phosphokinase (CPK) (U/L), thyroid-stimulating hormone (TSH) (mcU/mL), transaminases (AST and ALT) (UI/L), gamma-glutamyl-transferase (GGT) (U/L), lactate dehydrogenase (LDH) (mU/mL), triglycerides (mg/dL), serum iron (mcg/dL).

The local Ethical Committee (Fondazione IRCCS Ca’ Granda Ospedale Maggiore Policlinico) approved the protocol of this study (approval number 1789). The study was conducted in accordance with the provisions of the Declaration of Helsinki.

Assuming (1) a gender difference of at least 3 years in age of onset [36], (2) statistical significance at 0.05, and (3) power at 80%, a sample of 258 subjects (with at least 129 individuals for each subgroup) is considered adequate.

Statistical analyses were performed using the Statistical Package for Social Sciences (SPSS) for Windows (version 27.0). In our first step, we performed descriptive analyses for all participants (n = 555). The total sample was then divided in two groups according to gender (males/females). The male and female groups were compared using Student’s *t* test for continuous variables and chi-square tests for categorical variables (demographic and clinical variables are displayed in Table 1 and biological variables in Table 2). The p-values were adjusted for multiple comparisons using the Benjamini–Hochberg method. The variables that were statistically significant in preliminary analyses were then inserted into binary logistic regression models (enter method) with gender as dependent variable. Binary logistic regression modelling was performed subsequent to univariate analyses to ensure statistical robustness. We computed a model for continuous variables and a separate one for categorical variables. Finally, all statistically significant variables from the two above-mentioned binary logistic regression models were inserted in a new starting multivariable logistic regression model (enter method). The aim was to identify the variables independently associated with gender (Table 3). A similar statistical approach was employed by our research team in other analyses where a large pool of variables were potential covariates for the final model [37]. The goodness of fit of the models was assessed by the Omnibus and Hosmer–Lemeshow tests. The level of statistical significance was set at p ≤ 0.05 and confidence intervals at 95% for odds ratios were calculated.

**Table 1:** Demographic and clinical variables of the whole sample and of the two groups divided according to gender (males/females)

Variables	Total sample N = 555	Males N = 322 (58.0%)	Females N = 233 (42.0%)	p	q-FDR
Age (years)	43.40 (± 13.90)	40.62 (± 13.30)	47.24 (± 13.82)	**< 0.001**	**0.005**
Age at onset of schizophrenia (years) (missing = 236)	25.30 (± 8.70)	23.58 (± 7.32)	27.97 (± 9.95)	**< 0.001**	**0.005**
Duration of untreated illness (years) (missing = 409)	2.00 (± 4.73)	1.63 (± 4.01)	2.58(± 5.66)	0.237	0.329
Duration of illness (years) (missing = 237)	15.59 (± 12.88)	14.18 (± 11.92)	17.79 (± 14.02)	**0.015**	**0.049**
Duration of hospitalization (days) (missing = 270)	15.42 (± 13.85)	13.78 (± 11.66)	17.21 (± 15.60)	**0.047**	0.107
Number of cigarettes per day (missing = 421)	14.83 (± 15.47)	16.67 (± 15.27)	12.27 (± 15.52)	0.105	0.199
Number of lifetime hospitalizations (missing = 61)	5.97 (± 8.24)	5.44 (± 8.01)	6.65 (± 8.50)	0.377	0.439
Lifetime history of substance use disorders (missing = 134)	169 (40.1%)	135 (55.56%)	34 (22.08%)	**< 0.001**	**0.005**
Lifetime history of alcohol misuse (missing = 139)	87 (20.9%)	66 (25.20%)	21 (13.73%)	**0.006**	**0.026**
Lifetime history of poly-substance use disorders (missing = 143)	66 (16.0%)	53 (20.15%)	13 (8.72%)	**0.002**	**0.010**
Family history of psychiatric disorders (missing = 393)	83 (51.2%)	53 (56.38%)	30 (44.12%)	0.123	0.212
Family history of multiple psychiatric disorders (missing = 393)	28 (17.3%)	21 (22.34%)	7 (3.26%)	**0.045**	0.107
Family history of substance use disorders (missing = 399)	17 (10.9%)	14 (15.22%)	3 (4.69%)	**0.038**	0.098
Current smoking habit (missing = 226)	233 (70.8%)	167 (78.40%)	66 (56.90%)	**< 0.001**	**0.005**
Lifetime treatment with psychotherapy (missing = 274)	29 (10.3%)	11 (7.80%)	18 (12.86%)	0.164	0.246
Therapy with more than one antipsychotic drug at the time of hospitalization (missing = 287)	100 (37.3%)	56 (41.18%)	44 (33.33%)	0.184	0.262
Comorbidity with at least one psychiatric diagnosis (missing = 272)	51 (18.0%)	36 (25.71%)	15 (10.49%)	**0.001**	**0.005**
Comorbidity with more than one psychiatric diagnosis (missing = 272)	5 (1.8%)	5 (3.57%)	0 (0%)	**0.023**	0.073
Comorbidity with a diagnosis of personality disorder (Missing = 272)	5 (1.8%)	2 (1.43%)	3 (2.10%)	0.669	0.681
Presence of history of lifetime suicidal attempts (missing = 36)	58 (11.2%)	31 (9.72%)	27 (13.50%)	0.183	0.262
Number of lifetime suicide attempts (missing = 306)	0.24 (± 0.71)	0.18 (± 0.50)	0.33 (± 0.91)	0.129	0.216
Comorbidity with medical illnesses (missing = 209)	213 (61.6%)	108 (70.67%)	105 (62.50%)	0.727	0.727
Comorbidity with hypothyroidism (missing = 266)	19 (6.6%)	4 (2.82%)	15 (10.20%)	**0.011**	**0.039**
Comorbidity with diabetes (missing = 270)	25 (8.8%)	14 (9.86%)	11 (7.69%)	0.518	0.568
Comorbidity with hyper-cholesterolemia (missing = 32)	86 (16.4%)	46 (15.33%)	40 (17.94%)	0.427	0.481
Comorbidity with obesity (missing = 96)	20 (4.4%)	10 (3.95%)	10 (4.85%)	0.638	0.673
Comorbidity with multiple medical illnesses (missing = 267)	89 (30.9%)	40 (27.97%)	49 (33.79%)	0.285	0.369
Current treatment with statins (missing = 270)	6 (2.1%)	5 (3.52%)	1 (0.70%)	0.097	0.191
Current treatment with levothyroxine (missing = 270)	12 (4.2%)	3 (2.11%)	9 (6.29%)	0.079	0.167
GAF score (missing = 270)	34.46 (± 4.61)	36.26 (± 5.07)	32.66 (± 3.24)	**< 0.001**	**0.005**
PANSS score (missing = 270)	84.48 (± 8.75)	85.23 (± 9.60)	83.73 (± 7.78)	0.149	0.239

We reported means for continuous variables and frequencies for categorical ones. Standard deviations for continuous variables and percentages for categorical variables are reported into brackets. We have reported in bold statistically significant p or q-FDR values resulting from χ^2^ or unpaired Student’s t tests (p ≤ 0.05). q-FDR values from multiple comparison methods were based on Benjamini–Hochberg False Discovery Rate

*GAF* Global Assessment of Functioning, *PANSS* Positive And Negative Syndrome Scale

**Table 2:** Biological variables of the total sample and of the two groups divided according to gender

Variables	Total sample N = 555	Males N = 322 (58.0%)	Females N = 233 (42.0%)	p	q-FDR
Number of RBC (10^12^/L) (missing = 120)	4.79 (± 0.56)	4.9 (± 0.53)	4.59 (± 0.55)	**< 0.001**	**0.005**
MCV (fL) (missing = 366)	86.79 (± 9.46)	86.15 (± 10.3)	87.74 (± 8.02)	0.256	0.347
HB (g/dL) (missing = 115)	14.18 (± 1.63)	14.63 (± 1.57)	13.39 (± 1.44)	**< 0.001**	**0.005**
Number of WBC (10^9^/L) (missing = 120)	7.85 (± 2.65)	8.05 (± 2.85)	7.52 (± 2.22)	**0.044**	0.107
Number of lymphocytes (10^9^/L) (missing = 273)	2.34 (± 1.57)	2.47 (± 1.89)	2.13 (± 0.75)	0.079	0.167
Number of neutrophilis (10^9^/L) (Missing = 272)	4.61 (± 2.21)	4.69 (± 2.37)	4.48 (± 1.91)	0.430	0.481
NLR (missing = 283)	2.40 (± 1.85)	2.33 (± 1.56)	2.52 (± 2.27)	0.377	0.439
Number of PLT (10^9^/L) (missing = 374)	244.27 (± 74.5)	232.11 (± 72.54)	262.68 (± 74.14)	**0.007**	**0.028**
MPV (fL) (missing = 377)	11.99 (± 17.81)	10.67 (± 0.95)	13.97 (± 28.19)	0.328	0.406
PCHE (U/L) (missing = 386)	7822.95 (± 2160.63)	8017.00 (± 2220.88)	7534.72 (± 2050.09)	0.155	0.239
Total plasmatic proteins (g/dL) (missing = 337)	6.80 (± 0.60)	6.84 (± 0.51)	6.74(± 0.73)	0.273	0.362
Albumin (g/dL) (Missing = 332)	4.31 (± 0.46)	4.40 (± 0.39)	4.17 (± 0.54)	**< 0.001**	**0.005**
Bilirubin (mg/dL) (Missing = 133)	0.63 (± 0.44)	0.67 (± 0.49)	0.57 (± 0.35)	**0.033**	0.092
Uric acid (mg/dL) (Missing = 334)	5.14 (± 1.56)	5.27 (± 1.46)	4.94 (± 1.68)	0.123	0.212
Cholesterol (mg/dL) (missing = 135)	174.60 (± 41.90)	168.79 (± 41.78)	184.44 (± 40.37)	**< 0.001**	**0.005**
LDL (mg/dL) (missing = 298)	107.11 (± 33.40)	106.49 (± 35.49)	108.36 (± 28.87)	0.650	0.674
HDL (mg/dL) (missing = 286)	49.06 (± 14.95)	45.31(± 11.63)	56.50 (± 17.83)	**< 0.001**	**0.005**
Glycaemia (mg/dL) (missing = 118)	94.50 (± 26.07)	93.05 (± 25.88)	97.06 (± 26.29)	0.122	0.212
CPK (U/L) (missing = 375)	373.09 (± 895.20)	344.24 (± 501.74)	417.39 (± 1287.46)	0.593	0.638
TSH (mcU/mL) (missing = 313)	2.33 (± 2.56)	1.90 (± 1.24)	2.89 (± 3.56)	**0.008**	**0.030**
AST (UI/L) (missing = 166)	29.36 (± 28.76)	31.35 (± 32.69)	25.65 (± 19.96)	**0.028**	0.084
ALT (UI/L) (missing = 141)	27.88 (± 28.10)	29.58 (± 31.18)	24.81 (± 21.21)	0.090	0.183
GGT (U/L) (Missing = 132)	30.18(± 35.70)	31.44 (± 36.87)	28.06 (± 33.68)	0.346	0.420
LDH (mU/ml) (missing = 382)	223.37(± 100.80)	214.14 (± 89.83)	237.99 (± 115.27)	0.153	0.239
Triglycerides (mg/dL) (missing = 286)	108.98 (± 58.15)	111.56 (± 58.74)	104.26 (± 57.04)	0.326	0.406
Serum iron (mcg/dL) (missing = 386)	90.83 (± 38.86)	96.02 (± 38.94)	83.10 (± 37.70)	**0.034**	0.092

We reported means for all variables. Standard deviations are reported into brackets. We have reported in bold statistically significant p or q-FDR values resulting from unpaired Student’s t tests (p ≤ 0.05). q-FDR values from multiple comparison methods were based on Benjamini–Hochberg False Discovery Rate

*AST* aspartate aminotransferase, *ALT* alanine aminotransferase, *CPK* creatine phosphokinase, *GGT* gamma-glutamyltransferase, *HB* haemoglobin, *HDL* high density lipoprotein, *LDH* lactate dehydrogenase, *LDL* low density lipoprotein, *MCV* mean corpuscular volume, *MPV* mean platelet volume, *NLR* neutrophil to lymphocyte ratio, *PCHE* pseudocholinesterase, *PLT* platelets, *RBC* red blood cells, *TSH* thyroid-stimulating hormone, *WBC* white blood cells

**Table 3:** Final binary logistic regression model

Variables	B	S.E	Wald	p	OR	95% CI for OR
Presence of lifetime history of substance use disorders	1.241	0.483	6.606	**0.010**	0.277	0.177–0.435
GAF scores	− 0.282	0.073	14.376	**< 0.001**	0.754	0.653–0.871
Number of PLT	0.007	0.003	4.451	**0.035**	1.007	1.001–1.014
Bilirubin (mg/dL)	0.005	0.743	< 0.001	0.995	1.005	0.234–4.311
Total Cholesterol (mg/dL)	0.003	0.005	0.367	0.545	1.003	0.993–1.013

In this analysis the dependent variable was female gender versus male one

*B* regression coefficient, *CI* confidence interval, *GAF* Global Assessment of Functioning, *OR* odds ratio, *PLT* platelets, *S.E.* standard error of B, *Wald* Wald statistics

In bold statistically significant p (≤ 0.05)

## Results

The total sample included 555 patients. Of those, 322 were male (58.0%) and 233 were female (42.0%). The mean age was 43.40 ± 13.90 years, with a minimum age of 18 and a maximum of 84. Descriptive analyses and p-values of univariate analyses are reported in Table 1 (demographic and clinical variables) and Table 2 (biological variables).

With regard to the main antipsychotic treatment at the time of hospitalization, the information was available for 268 patients: 36 were not currently in treatment, while the others were under different antipsychotic treatment: specifically, 21 were treated with risperidone, 39 with haloperidol, 6 with paliperidone, 19 with olanzapine, 19 with quetiapine, 12 with aripiprazole, 16 with zuclopenthixol, 30 with clozapine, 2 with chlorpromazine, 1 with clotiapine, 9 with risperidone long-acting injection, 4 with olanzapine pamoate, 15 with zuclopenthixol decanoate, 26 with haloperidol decanoate, 7 with paliperidone long-acting injection, 3 with aripiprazole long-acting injection and 3 with fluphenazine decanoate. No statistically significant differences were found between genders regarding main antipsychotic treatment type at time of hospitalization (p > 0.05). In relation to psychiatric comorbidity, data were available for 283 patients: 232 had no comorbidity, 27 with substance use disorders, 1 with obsessive–compulsive disorder, 5 with autism spectrum disorder, 10 with eating disorders, 5 with personality disorders, and 3 with gambling disorder.

### Demographic and clinical variables

There were no significant differences between the two genders (p > 0.05) regarding several demographic and clinical variables (Table 1) including duration of untreated illness, number of lifetime hospitalizations, presence of family history of psychiatric disorders, presence and number of lifetime suicide attempts, comorbidity with medical disorders, and PANSS mean total scores. In contrast, males (compared to females) were, on average, younger at the time of hospitalization (t = 5.689, p < 0.001), had an earlier age at onset (t = 4.244, p < 0.001), a shorter duration of illness (t = 2.457, p = 0.015) and hospitalization (t = 1.999, p = 0.047), greater history of substance use disorders (χ^2^ = 32.978, p < 0.001), alcohol misuse (χ^2^ = 7.560, p = 0.006) and poly-substance use disorders (χ^2^ = 9.232, p = 0.002), showed more frequently a family history of multiple psychiatric disorders (χ^2^ = 4.005, p = 0.045) and of substance use disorders (χ^2^ = 4.310, p = 0.038), were more frequently smokers (χ^2^ = 16.810, p < 0.001), had a more frequent comorbidity with at least one psychiatric disorder (χ^2^ = 11.099, p = 0.001) and with multiple psychiatric conditions (χ^2^ = 5.199, p = 0.023), showed less often hypothyroidism (χ^2^ = 6.417, p = 0.011), and had higher mean GAF scores (t = 7.134, p < 0.001).

### Biochemical variables

As shown in Table 2, there were no differences between genders (p > 0.05) for several biochemical variables (e.g. MCV, number of lymphocytes and neutrophils, NLR, uric acid, triglycerides). In contrast, male patients (compared to female ones) had a higher number of RBCs (t = 6.006, p < 0.001), WBCs (t = 2.016, p = 0.044) and a lower number of PLTs (t = 2.751, p = 0.007), higher levels of HB (t = 8.226, p < 0.001), serum iron (t = 2.142, p = 0.034), albumin (t = 3.598, p < 0.001), bilirubin (t = 2.139, p = 0.033) and AST (t = 2.210, p = 0.028). However, levels of total cholesterol (t = 3.755, p < 0.001), HDL (t = 6.183, p < 0.001), TSH (t = 2.696, p = 0.008) were lower in males than females.

### Binary logistic regression

Regarding the binary logistic regression with continuous variables, the goodness-of-fit test (Hosmer and Lemeshow Test: χ^2^ = 5.933, p = 0.665) indicated that the model had a satisfactory goodness of fit, allowing for a correct classification of 76.6% of the cases. In addition, the model was significant overall (Omnibus test: χ^2^ = 45.274, df = 13, p < 0.001). Male patients (with respect to female ones) had higher mean GAF scores (p = 0.003), a lower number of PLTs (p = 0.008), higher levels of bilirubin (p = 0.042), and lower levels of total cholesterol (p = 0.025).

Regarding the binary logistic regression with categorical variables, the model had a satisfactory goodness of fit (Hosmer and Lemeshow Test: χ^2^ = 2.552, p = 0.863), allowing for a correct classification of 68.9% of the cases. Furthermore, the model was significant overall (Omnibus test: χ^2^ = 25.888, df = 7, p = 0.001). Of note, male patients are at higher risk for lifetime substance use disorder (p = 0.030), relative to females.

The final model (Table 3) had a satisfactory goodness of fit (Hosmer and Lemeshow Test: χ^2^ = 5.675, p = 0.684), allowing for a correct classification of 75.6% of the cases. In addition, the model was significant overall (Omnibus test: χ2 = 40.867, df = 5, p < 0.001). The presence of lifetime substance use disorders (p = 0.010), higher GAF mean scores (p < 0.001) and a lower number of PLTs (p = 0.035) were confirmed to be predictors of male gender.

## Discussion

The results of the present study confirm gender differences in the clinical and biochemical characteristics of subjects affected by schizophrenia. To our knowledge, this is one of the few studies that have addressed this topic.

With regard to clinical features, and in agreement with previous studies, men appear to have an earlier age at onset compared to women [1, 8]. This also explains our result indicating that women have a significantly older age of hospitalization. The impact of male hormones on dopamine pathways as well as the protective role of estrogens has been purported to explain the earlier onset of schizophrenia in males [38]. This hypothesis is supported by a study reporting higher serum levels of dehydroepiandrosterone sulfate, but lower levels of estrogens in male patients affected by schizophrenia (relative to healthy controls) [39]. The modulation of dopamine neurotransmission and reward circuitries caused by male hormones [40] would also explain the higher rates of lifetime substance use disorders and smoking in male schizophrenia patients (relative to females) [41].

Even though male patients had more psychiatric comorbidity at time of hospitalization, women reported lower GAF scores with respect to men. This result could be explained by the fact that women were older than males, on average, and had a longer duration of illness. Women also experienced longer hospitalizations and had more comorbidity with hypothyroidism, which supports these results. The vulnerability to thyroid dysfunction in our sample is also corroborated by the higher TSH levels in women relative to men. In general, thyroid dysfunction is more frequent in women than in men, though the reasons for this disparity is not clear [42]. As far as our findings, current literature reports more frequent hypothyroidism in patients with schizophrenia than healthy subjects [43], and variations of TSH levels according to the phase of the disorder [44]. However, gender differences in thyroid function in schizophrenia patients have not been extensively investigated until now. As the risk of hypothyroidism increases with age [45] and is associated with more severe course of psychotic disorders [46], the female preponderance of this condition in our sample could partially explain gender differences in social dysfunction and length of hospitalization; women in our sample were more prone to social dysfunction (relative to men) and had longer hospital stays.

The blood levels of several biochemical parameters resulted to be different according to gender. Some of these differences reflect what happens in the general population as the case of higher plasma levels of AST (also in relation to more frequent alcohol use disorders in male gender) [47] and of bilirubin [48] in males, lower levels of HB in females [49], a higher number of WBC in men than women till the age of 55 [50], a higher number of RBC in male patients than female ones [51] and vice versa for PLTs [52]. In contrast, in our sample women resulted to have higher total cholesterol plasma levels compared to men (confirmed by intermediate regression model), as reported in literature for younger women (aged 18–23 years); in contrast, for the age group ranging from 24–25 to 48–49 years old, higher cholesterol plasma levels were detected in men than in women [53]. Available literature displays a complex picture about the role of cholesterol on the course of different psychiatric disorders including schizophrenia [54]. Low levels of plasma cholesterol seem to be associated with suicide attempts [55] as a result of a change in neuronal membrane plasticity [56] and of the increase in the brain levels of oxysterols that are molecules favoring self-harm [57]. On the other hand, higher cholesterol plasma levels were found to be associated with clinical response to antipsychotics [58] and severity of depressive symptoms [59]. Response to antipsychotics [60] and the presence of depressive symptoms [19] are more frequent in women affected by schizophrenia than in men, in agreement with our findings. Of note, an article reported that, in schizophrenia patients, women have generally higher cholesterol plasma levels compared to men especially with advancing of age [61]. Similarly, the plasma levels of albumin resulted to be lower in female patients than male ones although no gender differences are reported in general population [62]. Albumin has antioxidant properties and current literature shows that patients affected by schizophrenia result to have lower albumin plasma levels than those with other psychotic disorders [63]. In addition, a Chinese study reported an inverse correlation between severity of depressive symptoms and levels of albumin in a sample of patients affected by schizophrenia in agreement with our results [64]. The inflammatory response seems to be an important factor associated with albumin leakage. Indeed, under inflammatory conditions, the transcapillary escape rate (TER) of albumin could increase by several folds. Possible direct or indirect mediators affecting TER of albumin were identified such as different pro-inflammatory cytokines including interleukin-2, interferon-alpha and interleukin-6 [65]. Interestingly, systemic immune activation and different inflammatory markers contribute to the onset and course of schizophrenia [66]. Women affected by schizophrenia were found to have higher levels of pro-inflammatory markers compared to men, thus supporting our findings about gender differences in albumin plasma levels [28].

## Conclusions

In summary, the results of our study confirm gender differences in clinical and biochemical parameters in patients affected by schizophrenia.

1. They indicate that women may have an initial clinical and biological advantage that is lost with advancing age [67], parallel to the predisposition to develop metabolic abnormalities perhaps driven by low-grade chronic inflammation [68];
2. show that men have an earlier age at onset and more lifetime substance use disorders;
3. can be translated in clinical practice with the choice of the best therapeutic approach according to gender.

With regards to the last point, clinicians could prescribe antipsychotics with less effect on metabolism for women or with less impact on the cytochrome system for men who frequently have comorbid substance use disorders. In fact, our data confirmed that women are more vulnerable to metabolic disorders; therefore, drugs with a lower metabolic impact should be prioritized in female patients. Regarding men, the high frequency of substance abuse is an important factor in determining treatment course, and should be considered in light of the potential pharmacokinetic and pharmacodynamic interactions with antipsychotics. Since heavy tobacco smoking is prevalent among patients suffering from schizophrenia, a modulation of the antipsychotic dosage may be required. It appears fundamental to underline that the choice of antipsychotic should consider not only gender, but also smoking status, substance abuse, family history, BMI, and, especially, age [58, 69]. With regard to women, an element to always keep in mind is menopause. In fact, pre-menopausal women appear to have a better response to antipsychotic treatment compared to post-menopausal ones, so that a dosage adjustment after menopause has to be considered good clinical practice [70]. This aspect confirms that estrogens play an important role in the onset and course of schizophrenia [27]. Of note, some studies report a beneficial effect of estrogen modulators (e.g. raloxifene) for ameliorating both negative and positive symptoms of schizophrenia in women [71, 72]. In addition, women after menopause are more vulnerable to osteoporosis that is worsened by hyperprolactinemia: compounds associated with a relevant increase of this hormone should be therefore prescribed with caution in post-menopausal women (e.g. amisulpride or risperidone) [73].

The following limitations are associated with this study: (1) although it was not possible to identify significant gender differences according to the type of treatment, the possibility that the last antipsychotic treatment may have influenced clinical and biological parameters cannot be excluded, (2) biochemical parameters may have been affected by the presence of substance use disorders or treatments for medical comorbidities, (3) biochemical parameters may have been affected by the administration of psychotropic drugs with metabolic impact, however, it seems interesting to underline that our sample presents metabolic parameters overlapping with those of the general population, (4) the fact of deriving data from clinical charts or patients’ interviews may make information less accurate, (5) the lack of a follow-up, (6) the impossibility to detect gender differences on specific symptoms such as disorganization or cognitive impairment, due to the unavailability of these data, (7) a lot of data are missing because some parameters are not routinely collected at the admission of patients in one or both centers. Being this a retrospective study, unfortunately it was not possible to collect all data at the admission of patients.

Further studies are needed to confirm gender differences in schizophrenia and translate this knowledge into clinical practice. These are foundational steps towards the implementation of precision medicine.

## Data Availability

The data of the findings presented in this study are available upon request to the corresponding author.
